# Microbiome-Aware Ecotoxicology of Organisms: Relevance, Pitfalls, and Challenges

**DOI:** 10.3389/fpubh.2020.00407

**Published:** 2020-08-21

**Authors:** Sébastien Duperron, Sébastien Halary, Alison Gallet, Benjamin Marie

**Affiliations:** ^1^Muséum National d'Histoire Naturelle, CNRS, UMR7245 Mécanismes de Communication et Adaptation des Micro-organismes, Paris, France; ^2^Institut Universitaire de France, Paris, France

**Keywords:** toxicology, microbiota, symbiosis, contaminants, resilience, environment

## Abstract

Over the last 15 years, the advent of high-throughput “omics” techniques has revealed the multiple roles and interactions occurring among hosts, their microbial partners and their environment. This microbiome revolution has radically changed our views of biology, evolution, and individuality. Sitting at the interface between a host and its environment, the microbiome is a relevant yet understudied compartment for ecotoxicology research. Various recent works confirm that the microbiome reacts to and interacts with contaminants, with consequences for hosts and ecosystems. In this paper, we thus advocate for the development of a “microbiome-aware ecotoxicology” of organisms. We emphasize its relevance and discuss important conceptual and technical pitfalls associated with study design and interpretation. We identify topics such as functionality, quantification, temporality, resilience, interactions, and prediction as major challenges and promising venues for microbiome research applied to ecotoxicology.

## Introduction: The Microbiome Is Relevant to Ecotoxicology

The significance of microbes to multicellular organisms is long documented. Because only a fraction of microorganisms can be isolated in culture, it is the advent of high-throughput sequencing technologies which ultimately revealed how diverse and numerically abundant they were. Microorganisms form complex symbiotic communities of eukaryotes, bacteria, archaea, and viruses referred to as the microbiome (1–3). Over the last 15 years, the microbiome has been a new frontier in Life Sciences (4), and microorganisms were shown to be involved and even necessary in many host functions including nutrition, defense, immunity, development, and behavior (2, 5, 6). A current paradigm assumes that most animals and plants harbor a microbiome (7). However, some species of comb jellies and nematomorpha, as well as certain life stages of insects including honeybee larvae, are apparently devoid of a microbiome, suggesting that this may be an over-simplification (8, 9). In humans, the microbiome may represent as many cells as the hosts and up to 1,000 times more genes, questioning the concept of individuality, and the limits of self (10, 11). The holobiont concept, referring to the entity formed by a host organism and its various microbial associates, arouse to encompass the complexity of hosts and their microbiome (12, 13). All these discoveries have fueled a “microbiome revolution” that increasingly spreads through all fields of life sciences, with extensions to behavioral and human sciences (14, 15).

**Figure 1:**
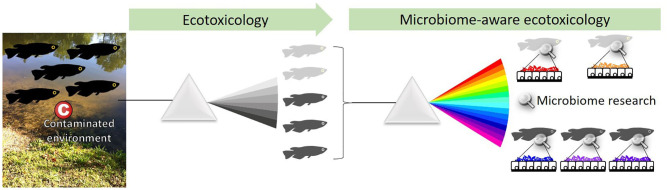
Ecotoxicology studies the effect of chemicals on organisms at the population level. A microbiome-aware ecotoxicology perspective acknowledges the importance of associated microorganisms in their hosts biology, at the level of individuals as well as populations. Indeed, the microbiome is interacting with hosts, environment, and contaminants and may be linked with health status (here, light vs. dark gray). The microbiome thus needs to be integrated as an element of the system, and protocols to investigate ecotoxicological effects at each level need to be adapted.

Recent research focuses mostly on the links between hosts and their microbiome, and the reciprocal influence they exert on each other, revealing its significance to host physiology, homeostasis, disease, health, and fitness (16). Interestingly, most members of the microbiome are located on epithelia (mucosa, skin...), i.e., animal or plant polarized tissues that separate the inside from the outside of the organism. Sitting at the interface between a host and its environment, epithelia and their associated microbiome are the hosts buffer and first line of defense against contaminants and environmental stressors (17). Deep-sea hydrothermal vent mussels are an example of this. They harbor bacteria located in the gill epithelium that oxidize hydrogen sulfide, a compound toxic to their hosts, and fix carbon that contributes hosts nutrition (18). Toxicology studies investigating the effects of chemical compounds on organisms examine the accumulation, bio-transformation, elimination, and effects in tissues, and are thus beginning to account for associated microbiome. This paper aims to emphasize the relevance, pitfalls, and promises of host-associated microbiome research for ecotoxicology and advocates for the emergence of a “microbiome-aware ecotoxicology” of multicellular organisms, i.e., an approach that fully incorporates the microbiome compartment as a dynamic interface interacting with host and environment (Figure 1).

## The Microbiome Responds to and Interacts With Contaminants

Data on effects of environmental contaminants on microbiomes has been published over the last years, with a focus on animal gut-associated bacteria (19–22). Published studies include controlled exposure of model organisms to various types of contaminants including pesticides, antibiotics, heavy metals, xenobiotics, or nanoparticles (23, 24). Bacterial community composition is typically assessed using the 16S rRNA sequence as a taxonomic marker that identifies Operational Taxonomic Units (OTUs), a commonly used proxy for species. The effect of exposures on OTU richness and diversity is then evaluated using multivariate statistics to test whether contaminants interfere with microbiome composition (25, 26). Many studies include complementary analyses on host parameters of toxicological relevance such as markers of the immune system, tissue histology, and developmental markers.

**Figure 2:**
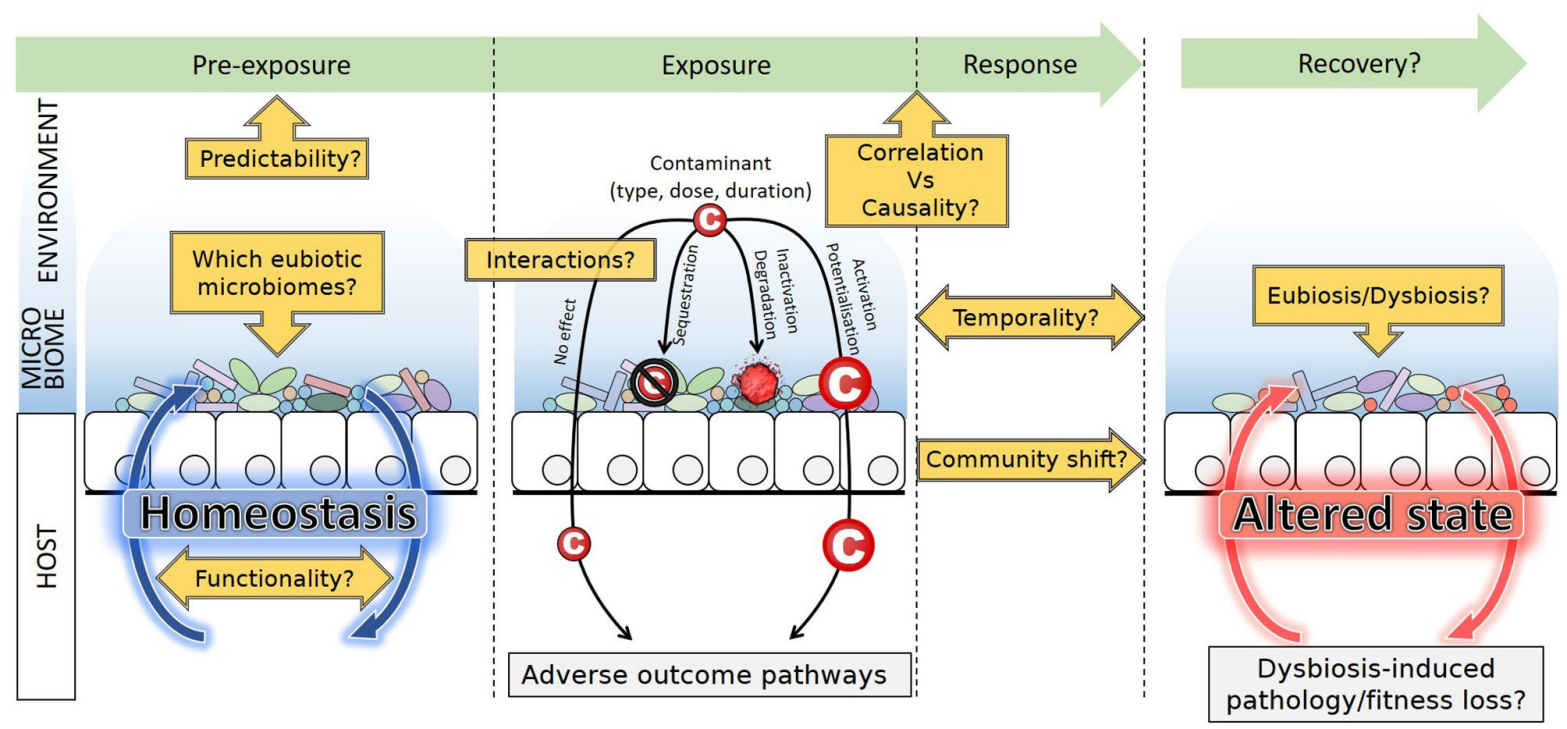
Sitting at the interface between environment and host, the microbiome may interact with contaminants. Sequestration, inactivation, and degradation mitigate potential effects on host health, while activation or potentialization reinforce the effect of contaminants. Microbiome composition, abundance and functions respond to exposure, and dysbiosis can occur. Post-exposure recovery leads to a new stable state, identical, or altered compared to the pre-exposure state. Future lines of research are emphasized. Although contaminants may alter the host health through adverse outcome pathways, dysbiosis itself may also induce pathology and fitness loss, difficult to disentangle from each other.

Besides descriptive studies that correlate exposures and microbiome variations, functional studies directly investigate the interactions between the gut microbiome and contaminants [Figure 2; (27)]. It has been shown that microbiomes of mammals guts are able to metabolize a wide range of xenobiotics (e.g., polycyclic aromatic hydrocarbons, polychlorobiphenyls, and nitrotoluenes) and could protect animals from deleterious effects (19). But the microbiome may also activate some compounds and mediate toxicity to hosts. For instance, the human colon microbiome was shown to convert polycyclic aromatic hydrocarbons into estrogenic metabolites with consequences on hormonal equilibrium (28); and the nephrotoxicity of melamine in rats results from its conversion into toxic cyanuric acid mediated by the bacterium *Klebsiella terrigena* (29). Many pharmaceutical drugs such as lovastatin or loperamide were also proved to be activated in the small intestine by bacteria-mediated biotransformations (30, 31). Alternatively, xenobiotics can also alter activities of the gut microbiome (23). Besides the obvious example of antibiotics, many molecules such as epoxiconazole or glyphosate, both pesticides, are known to induce shifts in microbiome compositions (32). Because the microbiome substantially responds and interacts with contaminants, it must now be considered a key player in toxicology.

## Producing and Interpreting Microbiome Data Relevant to Ecotoxicology

### Conceptual Pitfalls: Identifying a “Good” Microbiome and a “Good” Model Species

Ecotoxicology studies that address the microbiome rely on a microbial ecology background and need to consider the caveats associated with this discipline. Experimental investigations to date have focused mostly on bacteria. However, numerous studies have demonstrated the significance of Archaea and microbial Eukaryotes, including fungi, to host physiology (33, 34), and the key role of phages in regulating bacterial populations (35, 36). A comprehensive description of microbiome functioning requires all components to be accounted for (37). However, the lack of universal, easy-to-obtain markers for some groups, notably for viruses, still precludes the development of systematic analyses that require deep metagenomics, and specific expert analysis pipelines.

Besides, OTUs composition provides only a partial description of the real microbial diversity. Indeed, a single 16S rRNA-based OTU can encompass a diversity of distinct bacterial genotypes, potentially quite different in term of their respective functional phenotypes and responses to a stimulus (38, 39). The genus *Vibrio* for example includes strains with very different lifestyles, including commensals, light-producing mutualists of the squid *Euprymna scolopes*, and pathogens of numerous metazoans, that all display almost identical 16S rRNA sequences (40). The relative abundances and dynamics of these different phenotypes, can thus not be monitored using 16S rRNA.

Another major difficulty is the general lack of baseline knowledge regarding microbiomes of toxicology model species, for which very little-to-no data is available regarding wild populations (41–43). Besides, organisms used in tests are often sourced from rearing facilities, and have thus experienced domestication, a process documented to lead to massive changes in bacterial microbiome compositions. In vertebrates, changes include overall bacterial species richness decrease and shift in taxa abundances due to dietary, social, and environmental conditions of captivity [reviewed in (42, 44)]. Effects in other taxa are less documented and less clear-cut. In the silkworm for example, domestication is associated with higher bacterial diversity (45). The representativity of model species in ecotoxicology vs. their wild relatives thus remains to be evaluated in the light of their domestication history. Interestingly, humans are no exception to this trend, and gut microbiomes in industrialized societies greatly differ from the recent ancestral microbiome and from that of contemporary traditional populations (e.g., hunters-gatherers). Changes in diets, sanitation, and medical practices have led to a functional shift from fiber to mucus degraders, high frequency of antibiotic resistance, loss of particular taxa (e.g., Spirochaetes), and overall diversity decrease (46, 47). This is assumed to result in non-optimal microbiomes associated with increased risk of chronic diseases. Lack of knowledge, along with inter-individual variability (discussed below Technical Pitfalls: Performing the Right Experiment to Detect Effects), undermines the identification of the “normal,” balanced microbiome composition, i.e., the eubiotic state. This compromises the proper diagnosis of a dysbiosis (an “abnormal,” unbalanced) state upon exposure to contaminants (Figure 2). Indeed, although many factors can cause dysbiosis that may lead to health issues, it is not easy to establish what a healthy/eubiotic microbiome is (48). Recently, authors insisted that dysbiosis due to stressors is first of all the destabilization of the stable eubiotic state. Changes in abundances of certain beneficial taxa are evident signs of dysbiosis, but interestingly, increased inter-individual variability in microbiome composition could be another signature of dysbiosis (49).

In between the relative simplicity of most invertebrate-associated microbiomes in which a few OTUs are usually dominant [e.g., (50)] and the extreme complexity of mammal-associated microbiomes (with hundreds to thousands of OTUs), teleost fish and their tens to a few hundred bacterial OTUs offer an interesting intermediate, besides their relevance to the monitoring of aquatic ecosystems (51). Choosing a model thus involves addressing different levels of microbiome complexity, functionality, and domestication history. Whether current models in toxicology are relevant to microbiome-aware ecotoxicology studies needs to be evaluated.

### Technical Pitfalls: Performing the Right Experiment to Detect Effects

Most studies using controlled microcosms are monitoring various compartments that are potential sources of microbial diversity (e.g., food for animals, water for aquatic organisms). When scaling up to more holistic approaches such as mesocosms or the natural environment, potential sources of microorganisms dramatically increase, requiring the investigation of additional compartments (e.g., food, prays, parasites, water, particles, sediments).

Fifteen years of human gut microbiome research revealed the high level of intra- (between body regions or life stages) and inter-individual heterogeneity in community compositions (1, 52). Although less documented, high levels of intra- and inter-individual variation are reported in other taxa including fish [e.g., Atlantic cod, salmon, rainbow trout, zebrafish, (26, 51, 53–55)]. In the zebrafish for example, gut-associated communities become increasingly different from those in the environment, and inter-individual variation increases across development (56). Skin-associated communities are different on different body regions in the rainbow trout (55). These examples emphasize the importance of replication levels, and of addressing the exact same life stage and tissue region in all individuals.

Sex-differentiated responses to compounds are commonly reported in ecotoxicology studies, for example in medaka fish exposed to cyanotoxins (57). Sex also influences microbiome composition in various vertebrates, probably due to hormones and sex-specific immunity responses (58). It also affects microbiome responses. In lab-reared stickleback fed different diets, diet induced changes in some bacterial taxa abundances, but effects on bacteria in males were uncorrelated with effects observed in females, supporting that diet effects were clearly sex-specific (59). Authors measured similar sex-specific diet effects in mice and humans. Sex-specific effects on microbiome responses to contaminants are also documented. Exposure to silver nanoparticles was for example shown to modify the gut microbiome structure in male zebrafish, but not in females (60). Experimental design should thus carefully consider confounding factors of which sex is an important one.

## The Roads Less Traveled: Challenges in Microbiome-Aware Ecotoxicology

### Functionality and Integration

One major finding of the Human Microbiome Project was that despite high levels of intra- and inter-individual variation in the taxonomic compositions of bacterial communities, the functions they performed, as encoded by the metagenome, were highly conserved (1). Similar functions are thus performed by taxonomically distinct microorganisms. This concept known as functional redundancy is now recognized as key to the resistance and resilience of microbial communities (61, 62). Because of this, and the fact that closely related bacteria can display markedly different functionalities, community composition is not a reliable predictor of functions. Predictive tools for functional profiling based on composition [e.g., PICRUSt; (63)] thus suffer limitations, and identity and functions should ideally be investigated in tandem. Functional capabilities can be evaluated through metagenomic sequencing, but genes and functions that are expressed at specific time points are better evaluated by metatranscriptomic or metaproteomic approaches. Metabolomics, which map metabolites, are another important tool that profiles ongoing metabolisms, and thus informs functions (64, 65) although, as for all of the above, the improvement of databases supporting metabolite identifications will be critical (66). The integration of these approaches in multi-omics appears challenging, yet particularly promising for revealing the causal role of the microbiome and mechanisms involved in contaminants metabolism and toxic effects (67, 68). This will be key in integrating the microbiome in adverse outcome pathways (AOPs) and risk assessment [Figure 2; (22)].

### Quantification

Microbiome-aware ecotoxicology should identify contaminant threshold values relevant to microbiomes. Indeed, microbial communities may shift rapidly and non-linearly between contrasting alternative, more or less stable states provided some parameters reach threshold values. The existence of yet-undescribed tipping points is for example hypothesized to explain the existence of bimodal distributions of abundances of certain bacteria in the human gut (69, 70), and may be a trigger of dysbiosis. To identify tipping points in ecotoxicology, studies should examine dose-dependent responses and chronic exposure to low doses as done for toxicological effect on host traits, for example the determination of non-observable adverse effect limit (NOAEL).

Microbiome composition assessments also need to become more quantitative. Indeed, metabarcoding datasets produce taxa relative abundances tables, and their variations. In these, an increase in one group thus cannot be properly interpreted, as it may as well represent a lower decrease relative to other groups in a globally shrinking population. Bacterial densities in guts of distinct lineages of rainforest ants were for example shown to vary by orders of magnitude based on qPCR quantifications; interestingly, absolute abundance variations were better correlated with habitat (arboreal or terrestrial) and trophic position than actual community compositions (71). Antibiotics, which are reported to affect relative abundances in bacterial communities, act first by affecting the total number of bacteria present, as was clearly demonstrated for streptomycin and sancomycin, this being their major influence on the microbiome (72, 73). Absolute abundances are relevant to our understanding of the environment-host-microbiome continuum, and should thus be informed whenever possible, for example using quantitative PCR (74). Quantifying bacteria within organisms is however challenging, as demonstrated by the very different estimations of bacteria-to-human cell ratios found in the literature (75).

### Temporality and Resilience

The nature and amplitude of variations are important aspects of microbiome response to contaminants. Composition of communities and diversity indices are still the main endpoints of most studies. However, the dynamics of these variations during and after exposure are certainly as important. In humans, microbiome dynamics are individual-dependent (76). Dynamics inform resilience, evaluating whether variations have long-term effects on the microbiome, or whether it fully recovers and returns to a naive, pre-exposure stable state (Figure 2). Antibiotic exposure was for example shown to affect human gut bacterial communities for several months post-exposure, and similar effects can be expected with many contaminants (77, 78). Whether iterative exposure to some contaminants may lead to habituation, and thus become less influential to microbiomes over time, is also an important issue. Finally, how microbiome resilience itself may be affected by environmental factors (e.g., temperature, pH, interactions, seasonality) remains to be investigated.

### Interactions and Prediction

The holobiont is more than just the sum of its parts (13). With dozens-to-thousands distinct coexisting microbial taxa, and many more if phages are considered, an animal's gut or skin is a whole ecosystem in which multiple interactions among members and with the host influence its functioning. These as well as interactions with the environment, including the contaminants and microorganisms occurring there, need to be accounted for. For example, a new method coupling spatial imaging of metabolites and bacterial genotypes, MetaFISH, was developed to characterize interactions occurring between symbiotic bacteria and the gill epithelial cells of hydrothermal vent mussels. It allowed the identification of metabolites located at the host-symbiont interface on tissue sections at the micrometer scale (79). Such a method is promising to monitor small-scale interactions between contaminants, the microbiome and the host and further explore causality (68). Co-occurrence networks that are based on positive or negative correlations between the occurrence of microorganisms, functions, and environmental parameters also help in exploring interactions and formulating hypotheses [reviewed in (80)]. A strong relationship between the presence of a contaminant and that of certain bacterial taxa can suggest an ability to metabolize the former, which can then be tested (19). Changes in the network structure itself can indicate microbial successions in time series experiments or dysbiosis, and may support modeling approaches (81, 82).

## Conclusion: What Can the Microbiome Do for Ecotoxicology and Vice Versa?

By analogy with the famous essay written by Dobzhansky (83), it is tempting these days to suggest that “Nothing in Biology makes sense except in the light of the microbiome.” Ecotoxicology is no exception to this trend, and must not lag behind other disciplines that have embraced the microbiome revolution. However, the microbiome is not just another ecotoxicological endpoint, but a peculiar and complex biological compartment that exhibits its own ecological, metabolic, functional, and thus ecotoxicological rules (26). Instead, a microbiome-aware ecotoxicology of organisms needs to develop (Figure 1). This involves questioning, and not only transferring, classical toxicology protocols and model organisms' relevance to microbiome studies. Close cooperation between microbial ecologists and ecotoxicologists is needed. They have a lot in common: the complexity of microbiomes and their responses mirrors that of contaminants and their interactions; and both domains start with reductionist approaches, and strive to scale up to holistic approaches that encompass ecosystems full complexity and produce real-life-relevant data.

A major challenge is to move on from observing correlations to addressing causality, and ultimately explain processes, e.g., demonstrate mitigating effects of the microbiome at the population level in a given ecosystem. Repeatability is a key point, which involves inter-studies comparisons and meta-analyses for which tools are becoming available [e.g., Amplicon Sequence Variants for OTU clustering; (84)]. Microbiome features including taxa or functions may become bioindicators of contamination, as recently proposed in stream ecosystems (85). Modeling interactions between environment, contaminants, microbiomes, and hosts will become tractable, with a certain level of predictive power (86). No doubt the dialogue between disciplines will result in mutual enrichment, and will allow to make the most of the microbiome revolution applied to ecotoxicology.

## Author Contributions

SD, SH, AG, and BM have contributed to the writing of the manuscript and the production of figures. All authors have read and approved the final version.

## Conflict of Interest

The authors declare that the research was conducted in the absence of any commercial or financial relationships that could be construed as a potential conflict of interest.
